# Supplementary material for: Predicting maximum strain hardening factor in elongational flow of branched pom-pom polymers from polymer architecture
Source: Nat Commun. 2024 Apr 26;15:3545. doi: 10.1038/s41467-024-47782-8 (PMC11053115; doi:10.1038/s41467-024-47782-8)
Supplement: Supplementary file 1 — Supplementary Information [file 41467_2024_47782_MOESM1_ESM.pdf]

Supplementary Information to

Predicting Maximum Strain Hardening Factor in Elongational Flow of Branched Pom-Pom Polymers from Polymer Architecture

Max G. Schußmann[1], Manfred Wilhelm[1], Valerian Hirschberg*[1,2]

1) Institute for Chemical Technology and Polymer Chemistry, Karlsruhe Institute of Technology (KIT), Engesserstraße 18, 76131 Karlsruhe, Germany

2) Institute for Technical Chemistry, Technical University Clausthal, Arnold-Sommerfeld-Str. 4, 38678 Clausthal-Zellerfeld, Germany

Email: valerian.hirschberg@kit.edu

List of symbols

σ	Stress (Pa)
F	Force (N)
ω	Angular frequency (rad s^{-1})
T	Temperature (°C)
t	Time (s)
G_0	Plateau modulus (Pa)
$G_{N,s}^0$	Dilution modulus (Pa)
ϕ_b	Volume fraction of the backbone (-)
s_a	Number of entanglements of the arms (-)
s_b	Number of entanglements of the backbone (-)
s	Number of entanglements of a linear polymer (-)
$M_{w,b}$	Backbone molecular weight (kg mol^{-1})
$M_{w,a}$	Arm molecular weight (kg mol^{-1})
$Đ_b$	Backbone length dispersity (-)
$Đ_a$	Arm length dispersity (-)
q	Arm number (-)
$Đ_t$	Total dispersity of the pom-pom (-)
M_e	Entanglement molecular weight (kg mol^{-1}), $M_{e,PS}$ = 16.8 kg mol^{-1}
$\boldsymbol{S}$	Orientation tensor (-)

λ	Backbone stretch parameter (-)
s_c	Branch point withdrawal parameter (-)
τ	Relaxation time (s)
τ_b	Orientation relaxation time of the backbone (s)
τ_s	Stretch relaxation time of the backbone (s)
τ_a	Arm relaxation time (s)
τ_R	Rouse relaxation time of a linear polymer (s)
$\tau_{R,br.}$	Rouse relaxation time of branched polymers (s)
τ_l	Longest relaxation time (s)
τ_e	Rouse time of an entanglement (s)
ε	Hencky strain (-)
L_0	Initial sample length (m)
$L(t)$	Time dependent sample length (m)
$\dot{\varepsilon}$	Hencky strain rate (s^{-1})
ε_q	Hencky strain at maximum backbone stretch (-)
t_q	Time at maximum backbone stretch (s)
ε_f	Hencky strain to sample failure (-)
$\eta_E^+(t)$	Tensile stress growth coefficient (Pa s)
η_E	Extensional viscosity, $\eta_E = \eta_E^+(t)\vert_{t\to\infty}$ (Pa s)
η_0	Zero-shear viscosity (Pa s)
η_{LVE}^+	Linear viscoelastic envelope (Pa s)
f_c	Considère factor (-)
SHF	Strain hardening factor (-)
SHF_{max}	Maximum strain hardening factor (-)
η_{DE}	Steady state viscosity of the Doi-Edwards model (Pa s)
$\eta(\dot{\gamma}^{-1})$	Shear viscosity (Pa s)
$\dot{\gamma}$	Shear rate (s^{-1})
$\sigma_E^{Fracture}$	Maximum fracture stress of a C-C bond (Pa)
T_{ref}	Reference temperature of time-temperature superposition (°C)
G'	Storage modulus (Pa)

G'' Loss modulus (Pa)

δ Phase angle (°)

a_T Horizontal shift factor (-)

$Wi = \tau_l \dot{\varepsilon}$ Weissenberg number (-)

$De = \tau_l \omega$ Deborah number (-)

$Wi_{\delta,b,min}$ Weissenberg number of the phase angle minimum of the backbone (-)

$\delta_{b,min}$ Phase angle minimum of the backbone (°)

$Wi_{\dot{\varepsilon},frac}$ Weissenberg number where $\sigma_E^{Fracture}$ reaches plateau value (-)

$\dot{\varepsilon}_{frac}$ Strain rate at which fracture stress plateau is reached (s^{-1})

$M_{w,span}$ Span molecular weight of a pom-pom ($kg\ mol^{-1}$)

Supplementary Figures

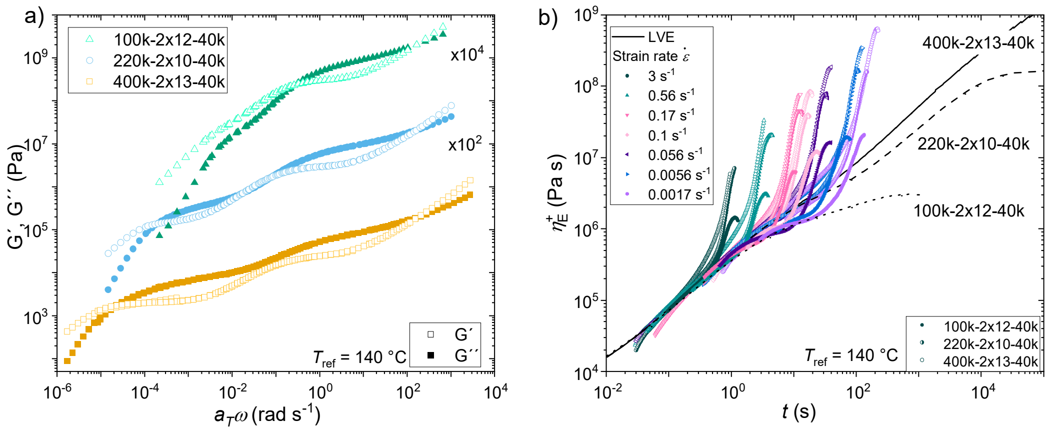

Supplementary Figure 1: a) Mastercurves of three pom-poms with different molecular weight of the backbone and arms of similar molecular weight and number. b) Measured tensile stress growth coefficient shown as a function of time for three pom-poms with varying molecular weight of the backbone.

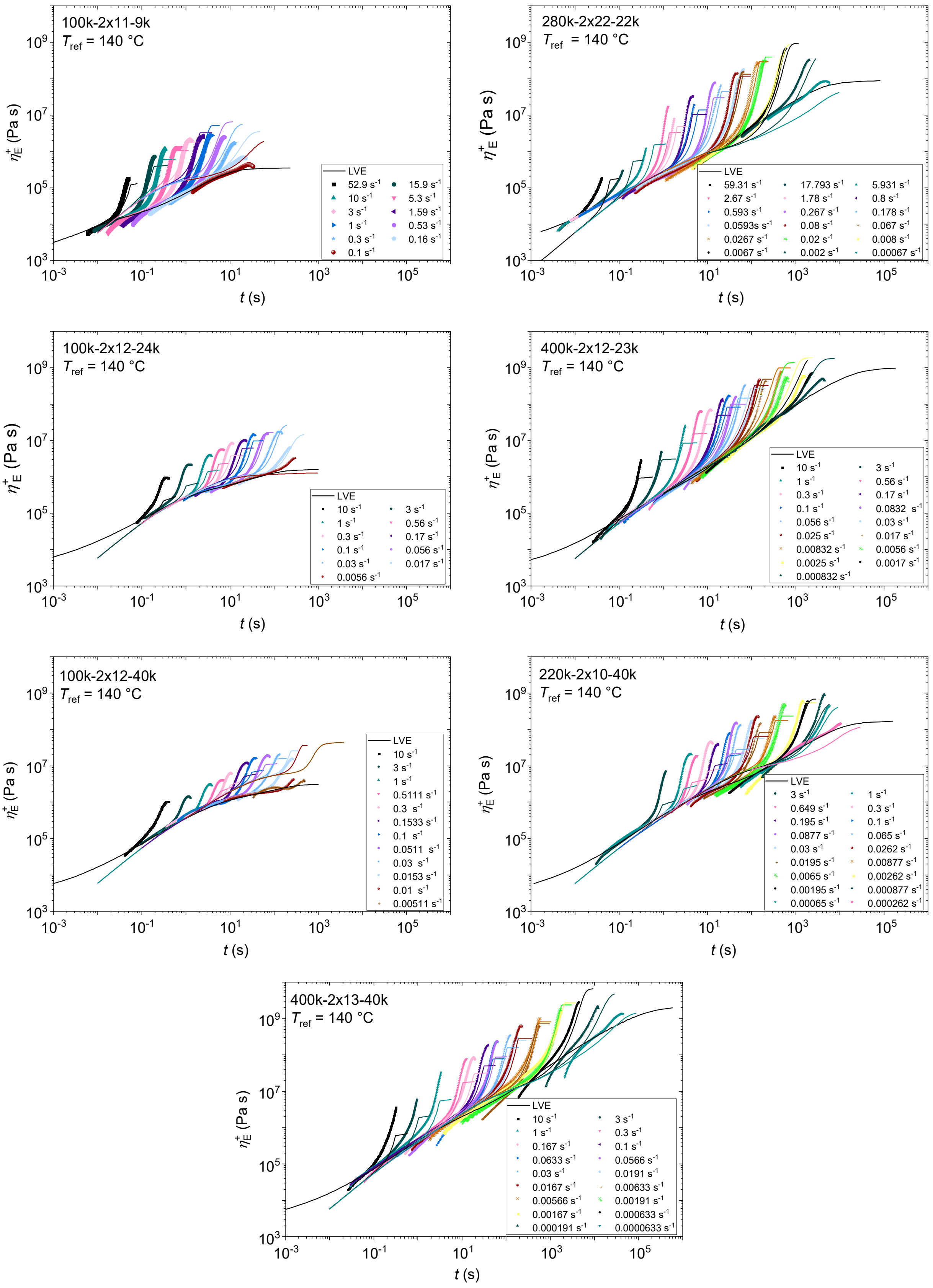

Supplementary Figure 2: Tensile stress growth coefficient as a function of time for the six pom-pom samples at a reference temperature of T_{ref}= 140 °C. Dots show experimental data, solid lines show simulated tensile stress growth coefficient by the pom-pom model calculated with the respective molecular parameters.

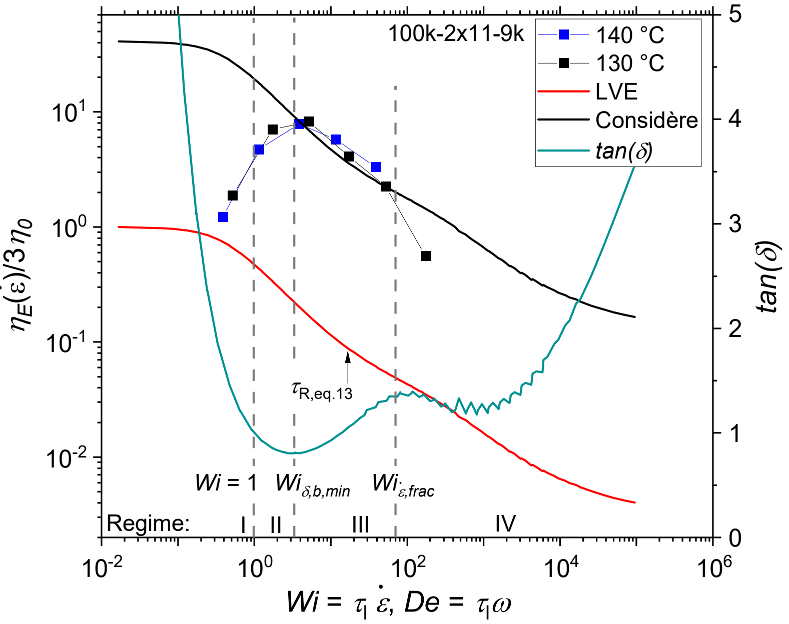

Supplementary Figure 3: Extensional viscosity η_E normalized to the zero-shear viscosity η_0 shown as a function of the Weissenberg number Wi and the phase angle $\tan\delta$ as a function of the Deborah number De of the pom-pom 100k-2x11-9k.

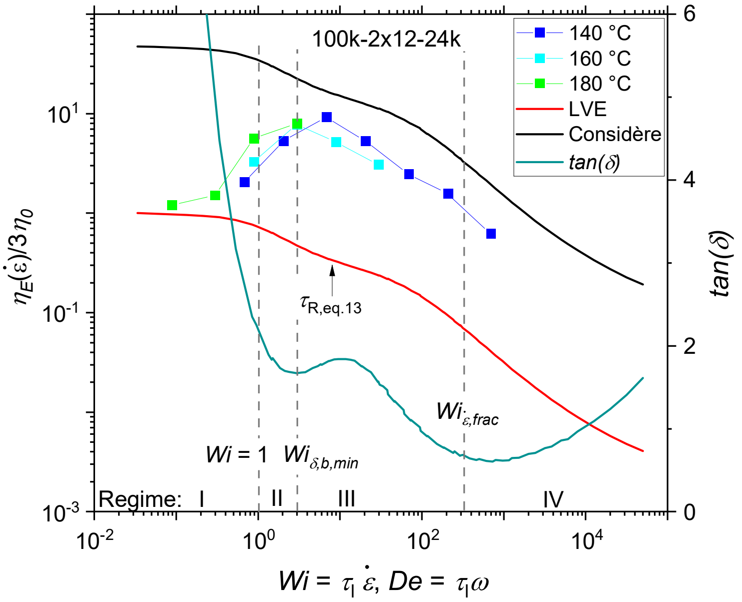

Supplementary Figure 4: Extensional viscosity η_E normalized to the zero-shear viscosity η_0 shown as a function of the Weissenberg number Wi and the phase angle $\tan\delta$ as a function of the Deborah number De of the pom-pom 100k-2x12-24k.

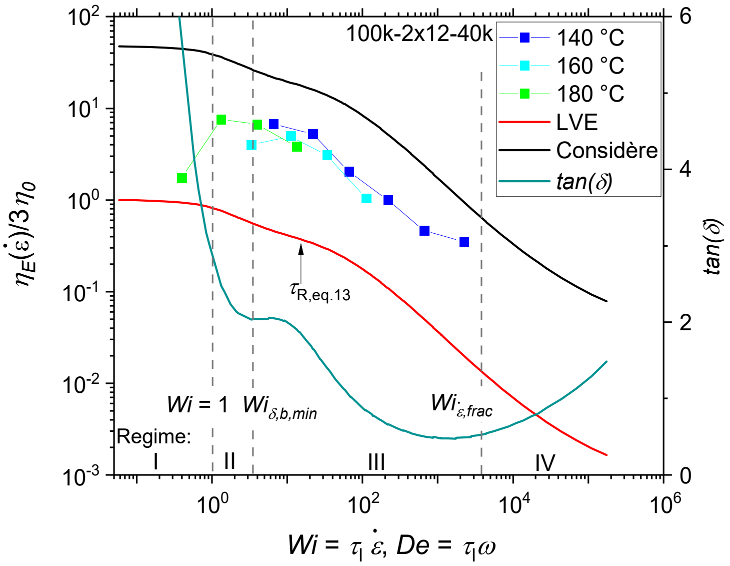

Supplementary Figure 5: Extensional viscosity η_E normalized to the zero-shear viscosity η_0 shown as a function of the Weissenberg number Wi and the phase angle $\tan\delta$ as a function of the Deborah number De of the pom-pom 100k-2x12-40k.

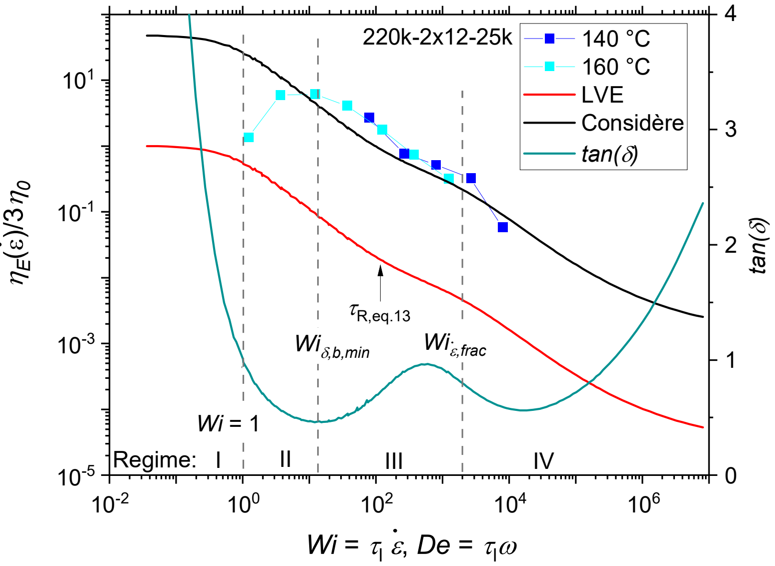

Supplementary Figure 6: Extensional viscosity η_E normalized to the zero-shear viscosity η_0 shown as a function of the Weissenberg number Wi and the phase angle $\tan\delta$ as a function of the Deborah number De of the pom-pom 220-2x12-25k.

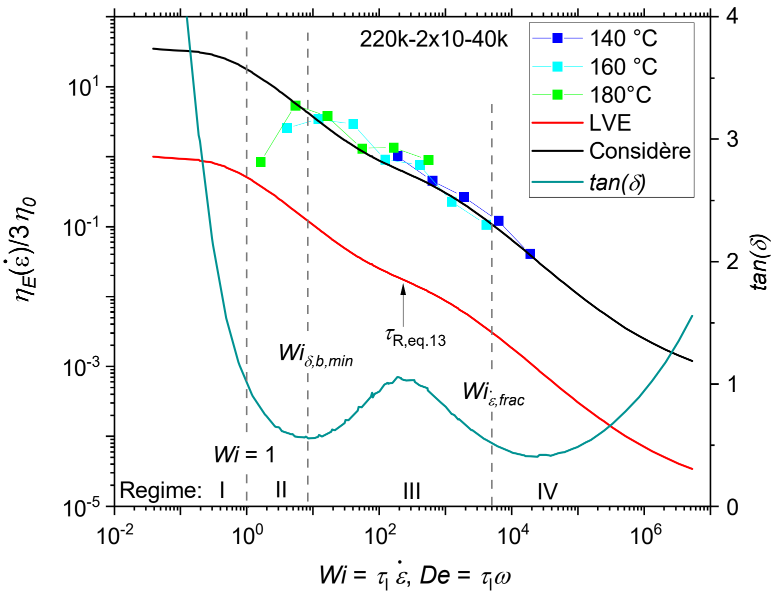

Supplementary Figure 7: Extensional viscosity η_E normalized to the zero-shear viscosity η_0 shown as a function of the Weissenberg number Wi and the phase angle $\tan\delta$ as a function of the Deborah number De of the pom-pom 220k-2x10-40k.

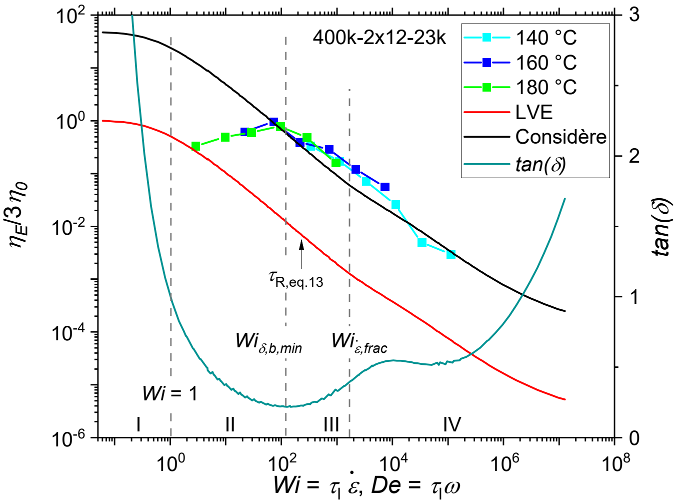

Supplementary Figure 8: Extensional viscosity η_E normalized to the zero-shear viscosity η_0 shown as a function of the Weissenberg number Wi and the phase angle $\tan\delta$ as a function of the Deborah number De of the pom-pom 400k-2x12-23k.

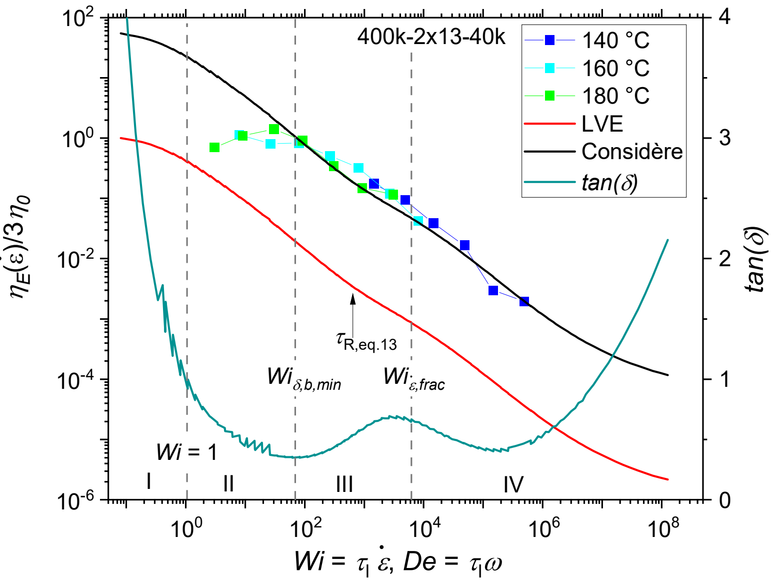

Supplementary Figure 9: Extensional viscosity η_E normalized to the zero-shear viscosity η_0 shown as a function of the Weissenberg number Wi and the phase angle $\tan\delta$ as a function of the Deborah number De of the pom-pom 400k-2x13-40k.

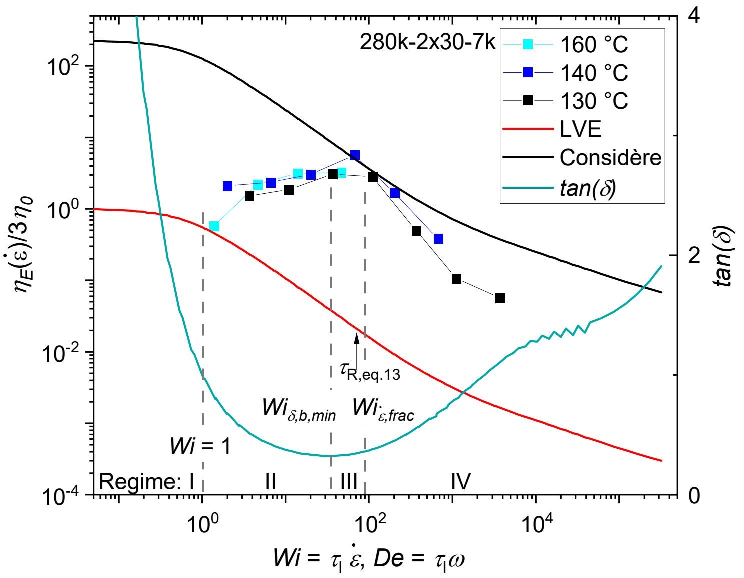

Supplementary Figure 10: Extensional viscosity η_E normalized to the zero-shear viscosity η_0 shown as a function of the Weissenberg number Wi and the phase angle $\tan\delta$ as a function of the Deborah number De of the pom-pom 280-2x30-7k.

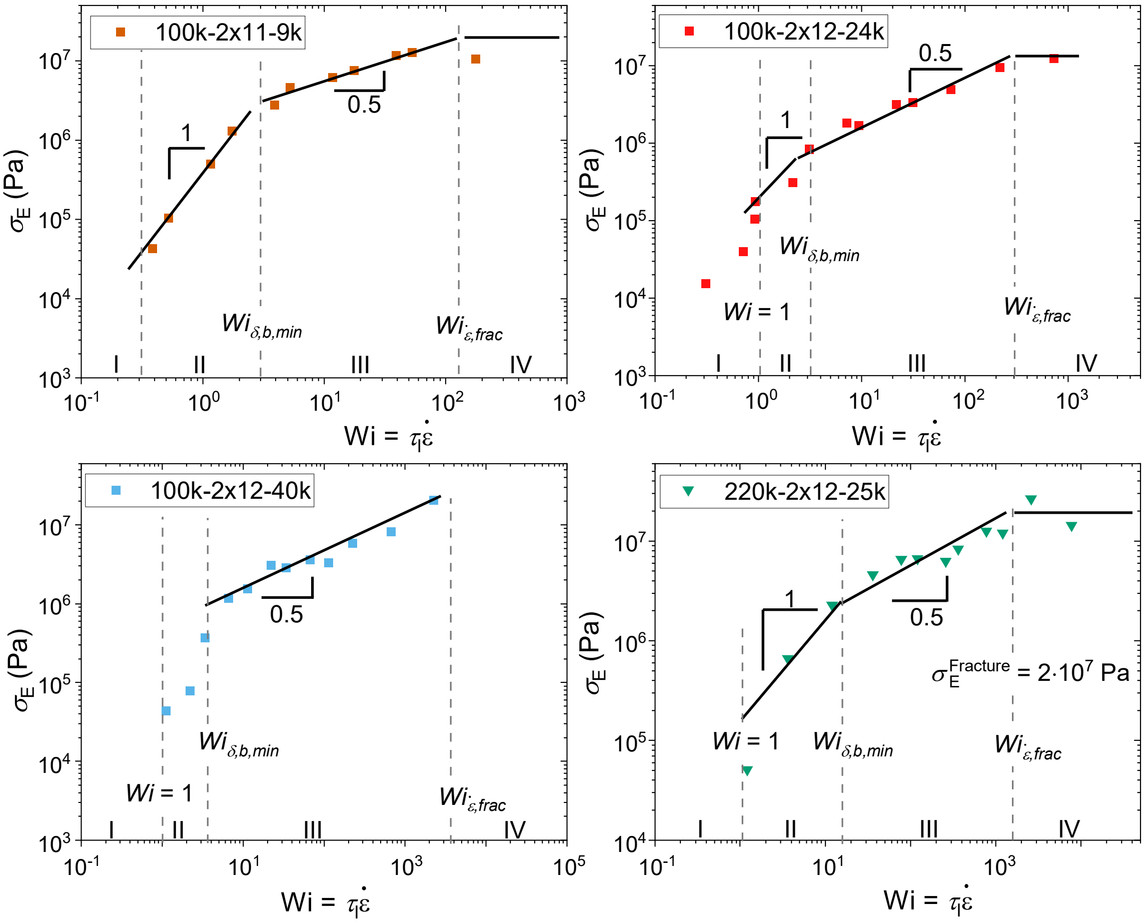

Supplementary Figure 11: Fracture stress $\sigma_E^{Fracture}$ as a function of the Weissenberg number Wi for all different pom-poms. Dashed lines indicate characteristic regimes. Solid lines are to guide the eye.

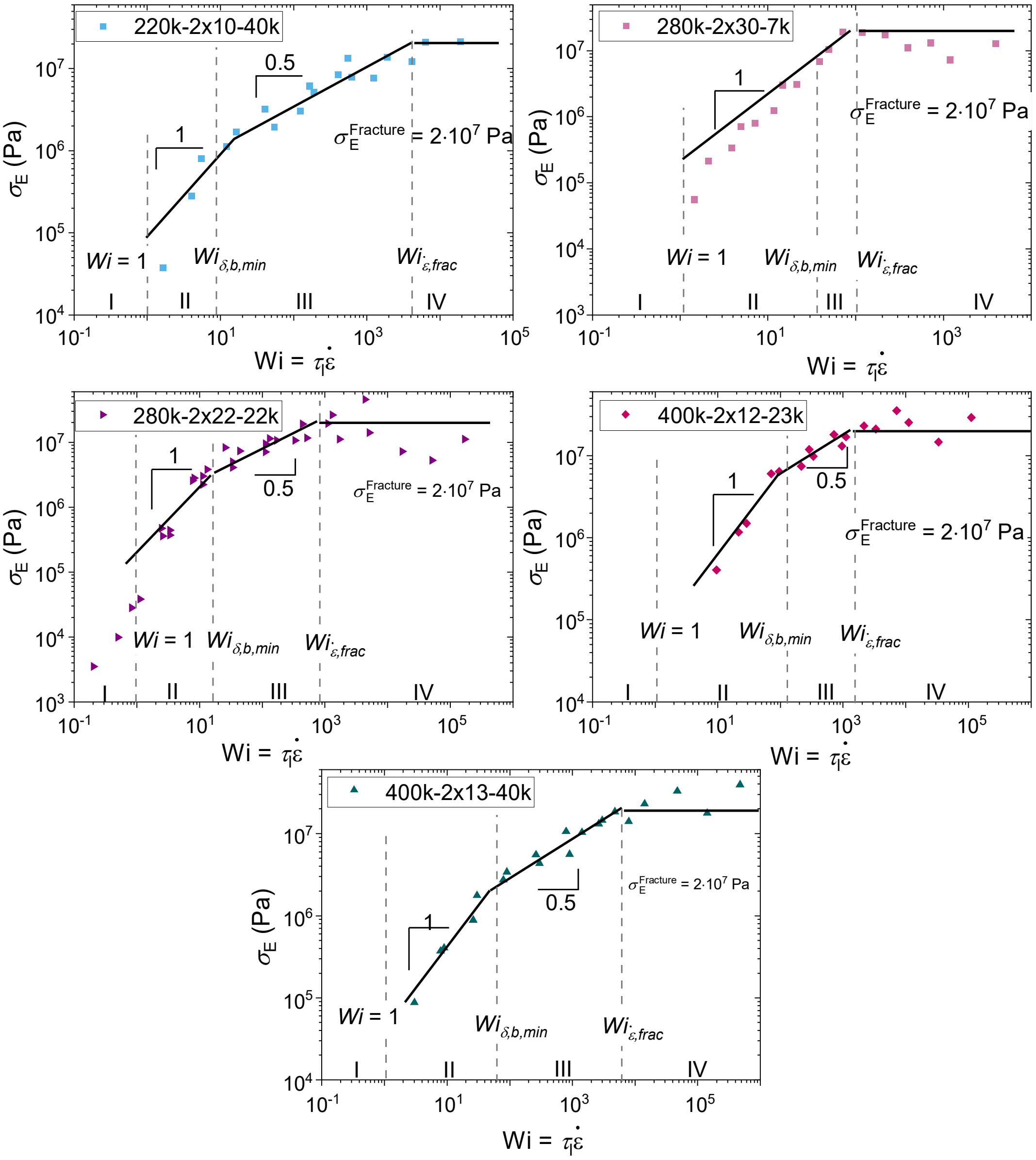

Continued Supplementary Figure 11: Fracture stress $\sigma_E^{Fracture}$ as a function of the Weissenberg number Wi for all different pom-poms. Dashed lines indicate characteristic regimes. Solid lines are to guide the eye.

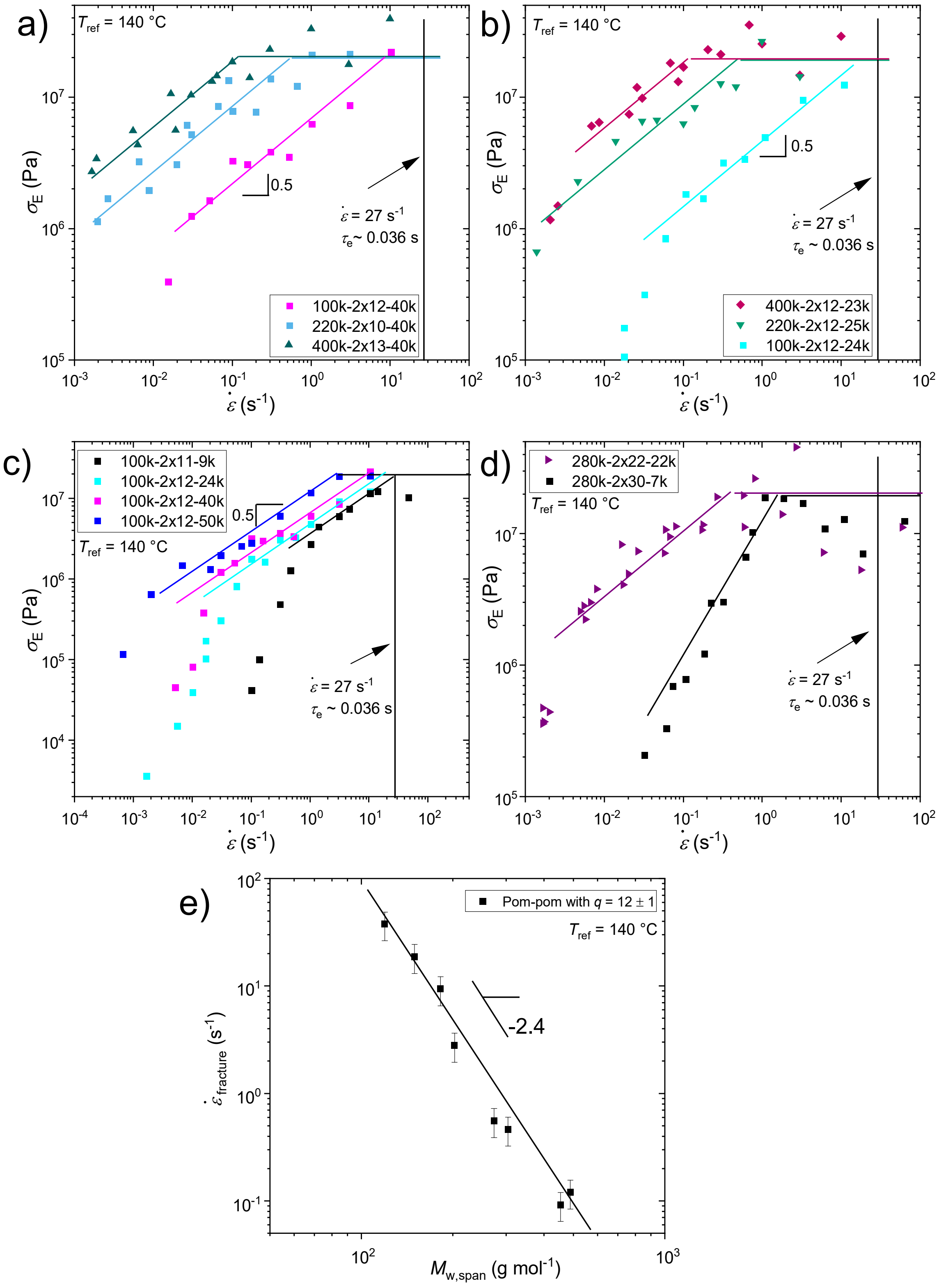

Supplementary Figure 12: a)-d) Fracture stress shown as a function of strain rate for four pom-pom series of different arm and backbone lengths. All strain rates are shown at a reference temperature of T_{ref} = 140 °C. e) Onset strain rate of the plateau stress as a function of the span molecular weight for pom-poms with $q = 12 \pm 1$.

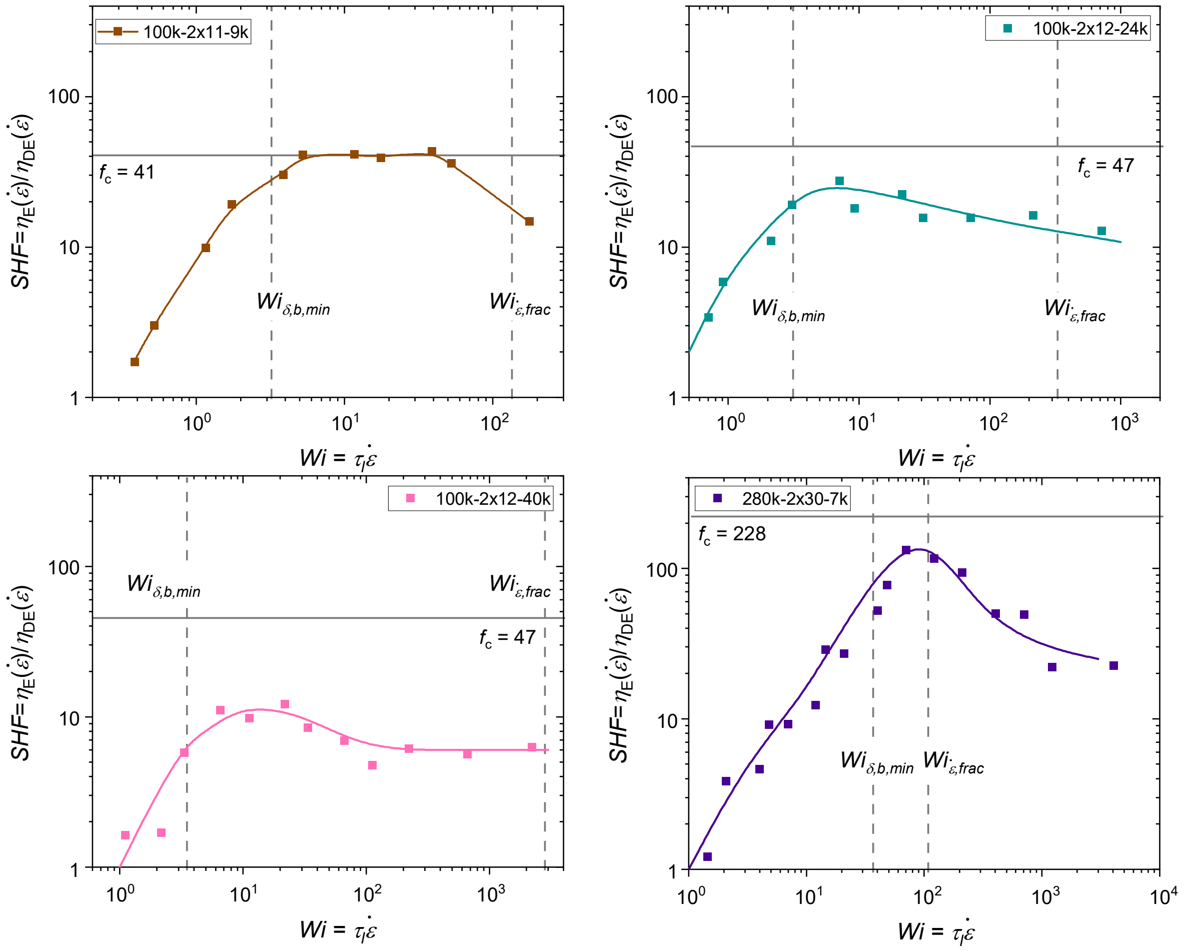

Supplementary Figure 13: Strain hardening factors SHF as a function of the Weissenberg number Wi for five pom-pom samples. The Considère factor f_c is shown for reference as the horizontal, grey line, calculated with the respective arm number of the sample. Vertical lines show the characteristic Weissenberg numbers.

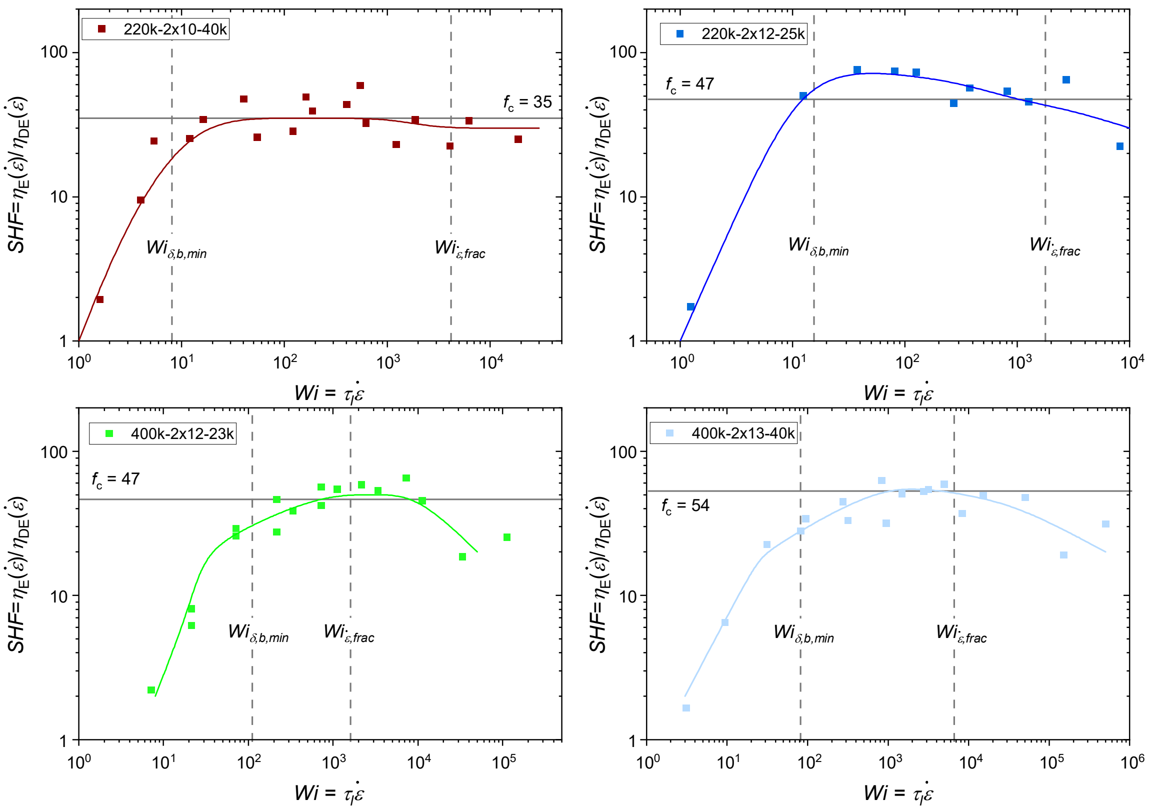

Supplementary Figure 13 continued: Strain hardening factors SHF as a function of the Weissenberg number Wi for four pom-pom samples. The Considère factor f_c is shown for reference as the horizontal, grey line, calculated with the respective arm number of the sample. Vertical lines show the characteristic Weissenberg numbers.

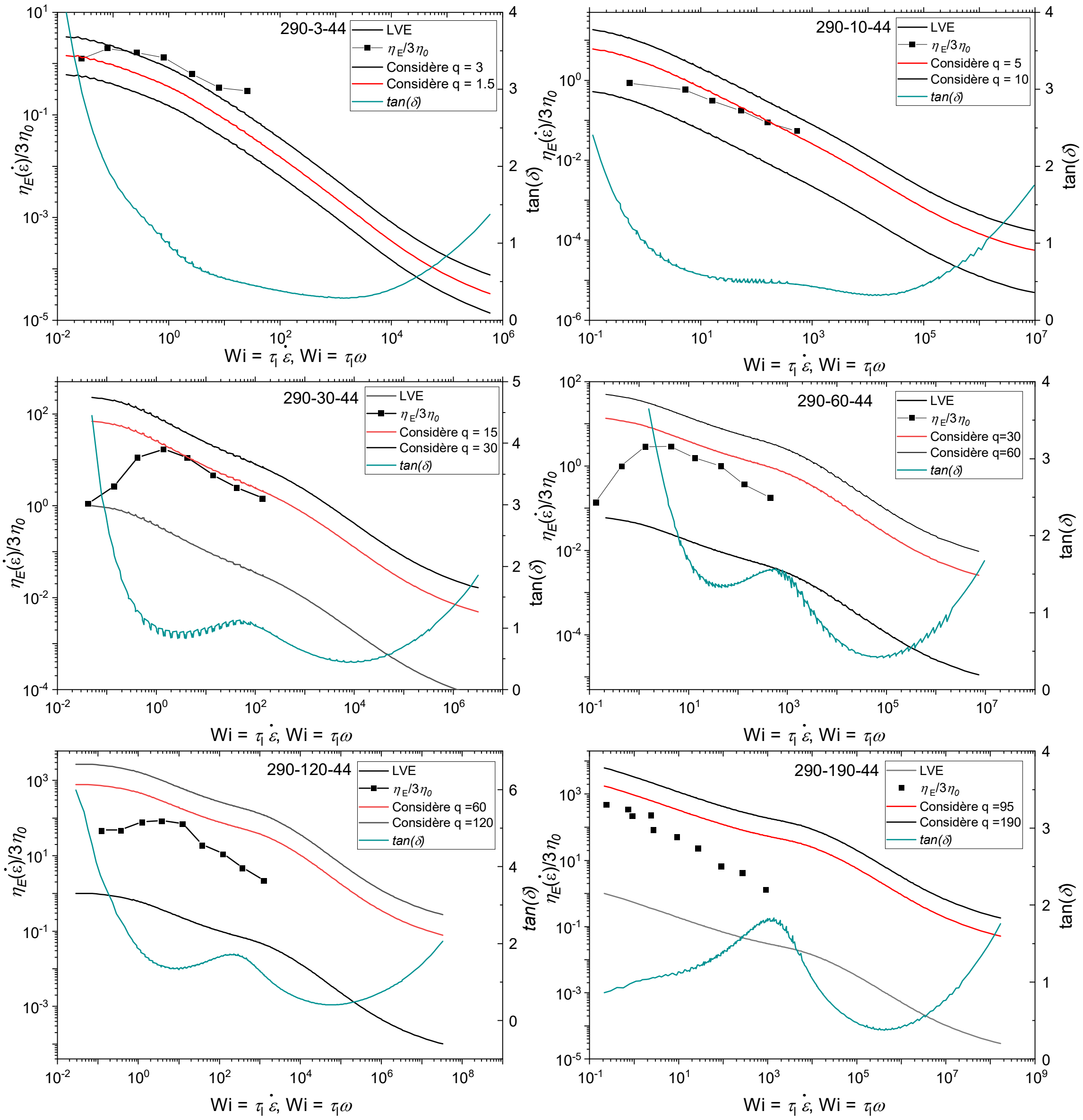

Supplementary Figure 14: Extensional viscosity η_E normalized to the zero-shear viscosity η_0 shown as a function of the Weissenberg number Wi and the phase angle $\tan\delta$ as a function of the Deborah number De of different combs. The combs are labelled with their backbone molecular weight $M_{w,b}$, number of branches k, and arm molecular weight $M_{w,a}$ resulting in $M_{w,b} - k - M_{w,a}$. For data see *Abbasi et al.*[46]

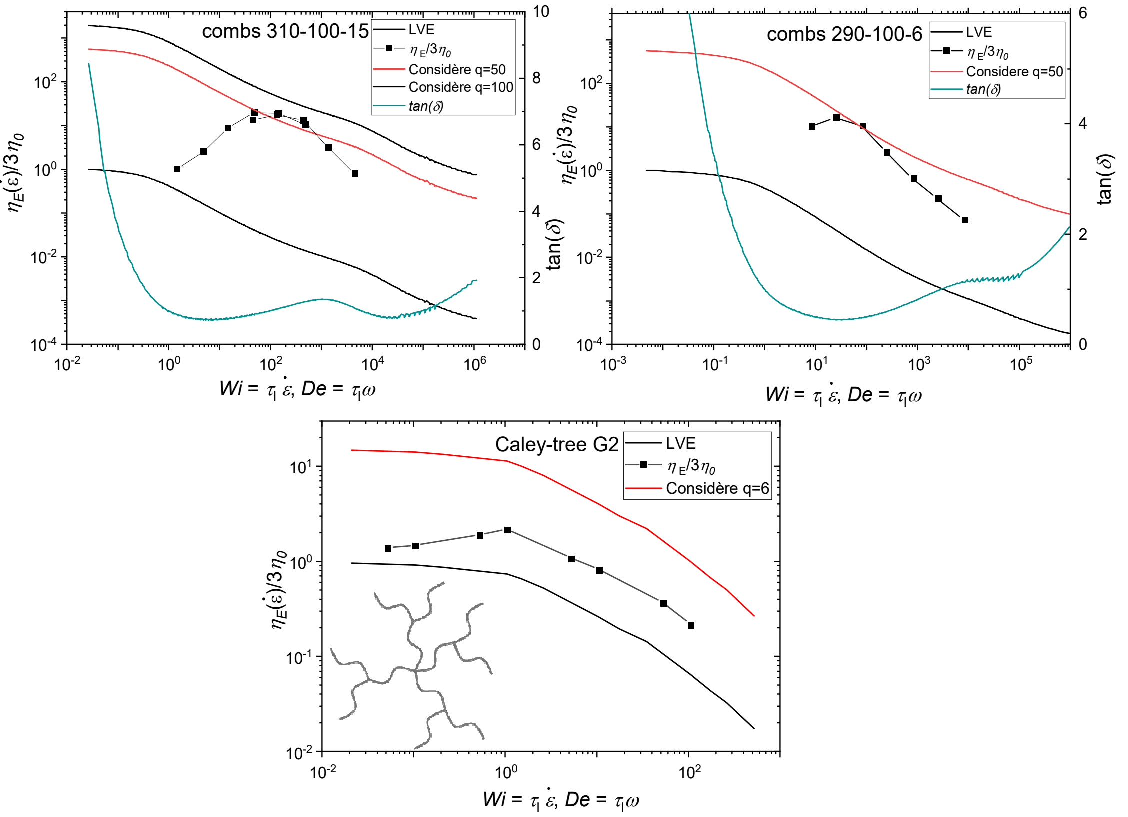

Supplementary Figure 15: Extensional viscosity η_E normalized to the zero-shear viscosity η_0 shown as a function of the Weissenberg number Wi and the phase angle $\tan\delta$ as a function of the Deborah number De of different combs and Cayley-tree samples. For Cayley-tree data see *van Ruymbeke et al.*[49]

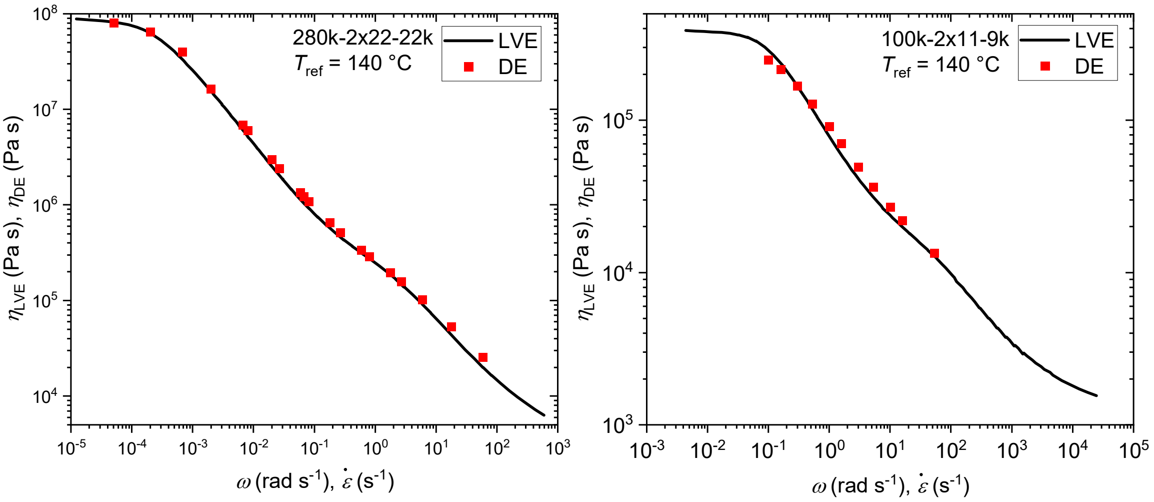

Supplementary Figure 16: LVE and the plateau viscosity of the DE model is shown as a function of angular frequency and strain rate, respectively.

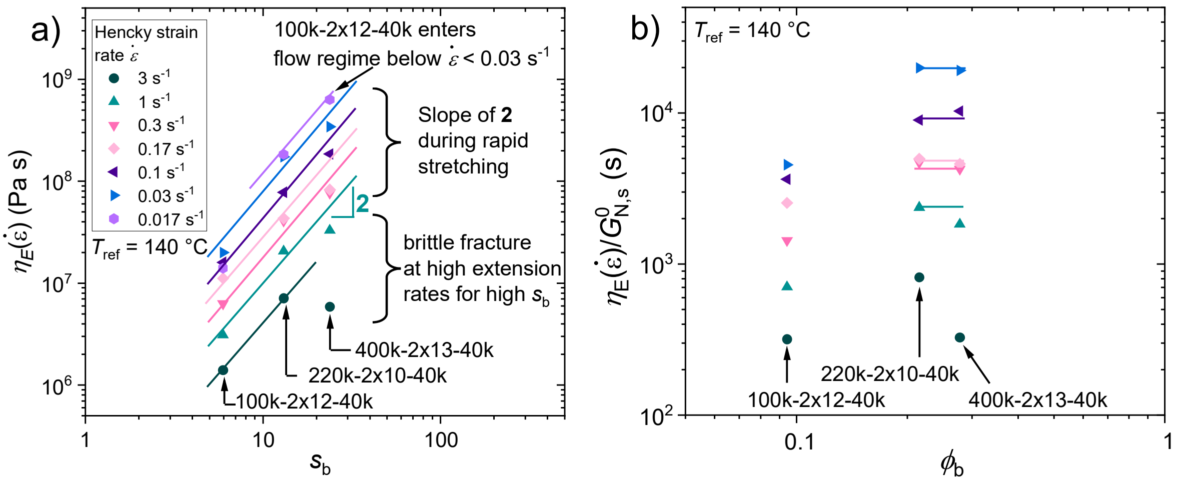

Supplementary Figure 17: a) Extensional viscosity η_E as a function of the number of entanglements of the backbone s_b for three pom-poms with varying molecular weight of the backbone. b) Extensional viscosity η_E normalized to the dilution modulus $G^0_{N,s}$ as a function of the volume fraction of the backbone ϕ_b. Selected strain rates between 3 s^{-1} and 0.0017 s^{-1} are shown to illustrate differences in the extensional behaviour of the three pom-poms.

ADDITIONAL METHODS

POLYMER SYNTHESIS

The pom-pom samples were synthesized in a three-step procedure using anionic polymerization and post-polymerization modification, as shown in Supplementary Figure 18.[39,61] In the first step, a poly-isoprene-*b*-styrene-*b*-isoprene (ISI) triblock copolymer was synthesized by sequential monomer addition using *s*-BuLi as initiator in cyclohexane. The polyisoprene (PI) blocks are used as branching points and are kept much shorter compared to the polystyrene (PS) block (> 1:15:1 mol%), as illustrated in Supplementary Figure 18. In the second step, the obtained ISI triblock was functionalized by epoxidation of the double bonds of the *cis*-1,4-polyisoprene units using hydrogen peroxide and formic acid. In the third step, living polystyrene chains were synthesized and then grafted onto the epoxidized ISI triblock, resulting in the pom-pom topology, with full control over all molecular parameters and enabling high arm numbers.

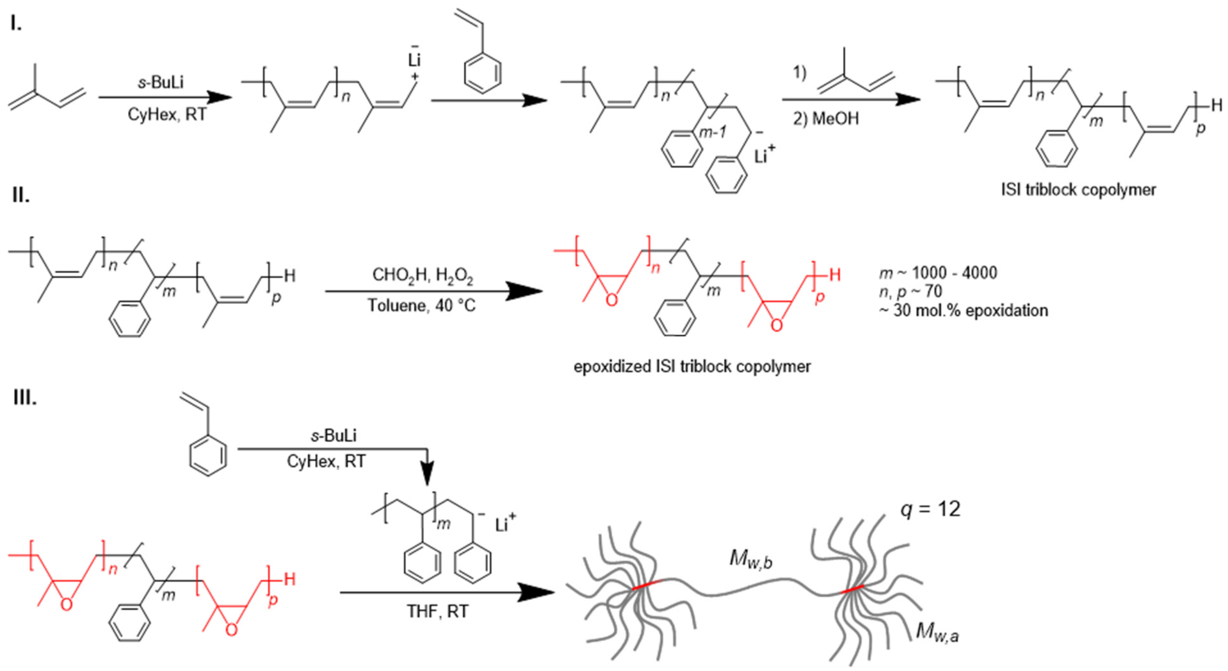

Supplementary Figure 18: Synthetic scheme of the synthesis of polystyrene pom-poms: I. Polymerization of the ISI triblock copolymer, II. Epoxidation of the ISI triblock and III. Grafting onto the epoxidized ISI triblock with living polystyrene chains.

Amounts of reagents were adjusted according to the desired molecular parameters q, $M_{w,a}$, and $M_{w,b}$. All samples and their molecular parameters are determined by size exclusion chromatography and in line multi angle laser light scattering (MALLS) listed in Supplementary Table 1, further synthetic details can be found in earlier work.[39,40,46,62,63] In the here presented work, we synthesized two series of pom-poms with similar arm molecular weight ($M_{w,a}$ = 24 or 40 kg mol^{-1}) and number of arms (q_{24k} = 12, q_{40k} = 10 – 13) and increasing molecular weight of the backbone ($M_{w,b}$ = 100 – 400 kg mol^{-1}) referred to by the length of the arms as 24k-series and 40k-series as shown in Supplementary Table 1.

Supplementary Table 1: Molecular parameters of the synthesized PS pom-poms. The molecular weight of the arms is $M_{w,a}$, q_a the number of arms per star/pom and $M_{w,b}$ is the molecular weight of the backbone. The polydispersity of the backbone, arms, and total pom-pom are $Đ_b$, $Đ_a$ and $Đ_t$, respectively. The number of entanglements of the backbone s_b and the sidechains s_a are calculated using an entanglement molecular weight of M_e = 16.8 kg mol^{-1}.[41] ϕ_b is the volume fraction of the backbone. [a] Measured with SEC-MALLS. Arm numbers of samples 220k-2x12-25k and 400k-2x12-23k are recalculated based on new SEC-MALLS measurements compared to previous work. SEC-MALLS revealed slightly higher arm numbers than previously determined by SEC-DRI.

Sample	$M_{w,\mathrm{b}}$ (kg/mol)	s_b	$Đ_b$	$M_{w,\mathrm{a}}$ (kg/mol)	s_a	$Đ_a$	q	$M_{w,\mathrm{t}}$[a] (kg/mol)	$Đ_t$	ϕ_b
**pom-pom $M_{w,\mathrm{b}} - 2\mathrm{x}q - M_{w,\mathrm{a}}$**										
**24k-series**										
100k-2x12-24k	100	5.95	1.05	24	1.42	1.05	2x12	600	1.18	0.15
220k-2x12-25k	220	13.1	1.06	25	1.49	1.03	2x12	808	1.08	0.27
400k-2x12-23k	400	23.8	1.10	23	1.37	1.03	2x12	932	1.14	0.42
**40k-series**										
100k-2x12-40k	100	5.95	1.05	40	2.38	1.08	2x12	1060	1.16	0.09
220k-2x10-40k	220	13.1	1.06	40	2.38	1.03	2x10	1014	1.09	0.22
400k-2x13-40k	400	23.8	1.10	40	2.38	1.03	2x13	1338	1.15	0.28
**Additional samples**										
100k-2x11-9k	100	5.95	1.05	9	0.54	1.02	2x11	298	1.13	0.34
280k-2x30-7k	280	16.7	1.04	7	0.42	1.03	2x30	694	1.16	0.40
280k-2x22-22k	280	16.7	1.04	22	1.31	1.08	2x22	1248	1.15	0.22

Materials: Styrene (99% extra pure, stabilized, Sigma-Aldrich) was stirred over calcium hydride (CaH_2, 92% Fisher Scientific) over night, degassed by three freeze-pump-thaw cycles, and freshly distilled prior to use. Isoprene (98%, stabilized, VWR) was stirred in an ice bath over *n*-butyllithium (2.5 M in cyclohexane, Sigma-Aldrich). As soon as the solution turned slightly yellow, isoprene was distilled into ampules. Cyclohexane (99%, Fisher Scientific) was purified by stirring over polystyrenyllithium, degassed by three freeze-pump-thaw cycles, and freshly distilled prior to use. Tetrahydrofuran (THF, 99.5%, Roth) was distilled from CaH_2, stored over sodium/benzophenone, degassed by three freeze-pump-thaw cycles, and freshly distilled before use. Methanol (>99%, Fisher Scientific) was degassed by three freeze-pump-thaw cycles. 1,4-dioxane (>98%, Fisher) is purified by distillation under reduced pressure before use. Toluene (>99%, Roth), hydrogen peroxide (H_2O_2, 30 wt %, Acros Organics), formic acid (98%, Roth), *s*-butyllithium (*s*-BuLi, 1.4 M in hexane, Aldrich), sodium (>99%, Merck), THF (HPLC grade, Fisher), and benzophenone (Sigma-Aldrich) was used as received.

The SEC instrument is an Agilent 1200 series equipped in a four-detector configuration with UV, multiangle laser-light scattering (MALLS, SLD7000/BI-MwA (PSS, Brookhaven Instruments)), viscometer and a DRI detector (Polymer Standard Service, PSS, Germany), using an autosampler for sample injection. The columns (SDV-Lux 5 µm (guard column), SDV-

Lux-1000 Å, SDV-Lux 100,000 Å, PSS) were calibrated with monodisperse linear PS standards with weight-average molecular weights, between 476 and $2.5 \cdot 10^6$ g mol^{-1}. HPLC grade THF was used as the eluent with a flow rate of 1 ml min^{-1}. 100 µl were injected for each measurement. dn/dc values were calculated based on ratio of the blocks (mol.%) as determined by ^{1}H NMR.

^{1}H NMR spectra were obtained in deuterated chloroform at 25 °C using a 400 MHz Bruker Avance III Microbay spectrometer.

All polymerization steps were conducted under inert Schlenk conditions.

1) Synthesis of the backbone: 0.73 mL isoprene (0.50 g, 7.33 mmol) and 0.072 mL s-BuLi solution (1.4 M, 0.1 mmol) are added to 100 mL of freshly distilled cyclohexane at room temperature. The polymerization is allowed to proceed overnight. 9.9 mL styrene (9.00 g, 0.086 mol) is added, and the polymerization is allowed to proceed overnight. 0.73 mL isoprene (0.50 g, 7.33 mmol) is added, and the polymerization is allowed to proceed overnight and then terminated by 1 mL MeOH. The polymer is precipitated from MeOH and freeze-dried from 1,4-dioxane solution.
2) Epoxidation: 10 g ISI triblock copolymer (10wt%. PI) is dissolved in 100 mL toluene and heated to 40 °C. 0.41 mL formic acid (0.50 g) is added and 1.77 g H_20_2 solution (1.59 mL, 0.016 mol) was added dropwise. The reaction mixture was stirred for 45 mins at 40°C. The organic phase was washed three times with 100 mL water.
3) Synthesis of the arms: 19.8 mL styrene (18 g, 0.173 mol) is added to 200 mL of cyclohexane and initiated by 0.64 mL *s*-BuLi solution (1.4 M, 0.90 mmol) at room temperature. The polymerization is allowed to proceed overnight.
4) Grafting reaction: 3.00 g dried backbone is dissolved in 100 mL THF. The solution of living arms (18 g in 200 mL, Ratio arms to backbone 30:1) are added and the reaction is stirred for 16 h at room temperature. Residual arms are terminated with 1 mL degassed MeOH. The polymer is precipitated from MeOH and fractionated with MeOH/THF.

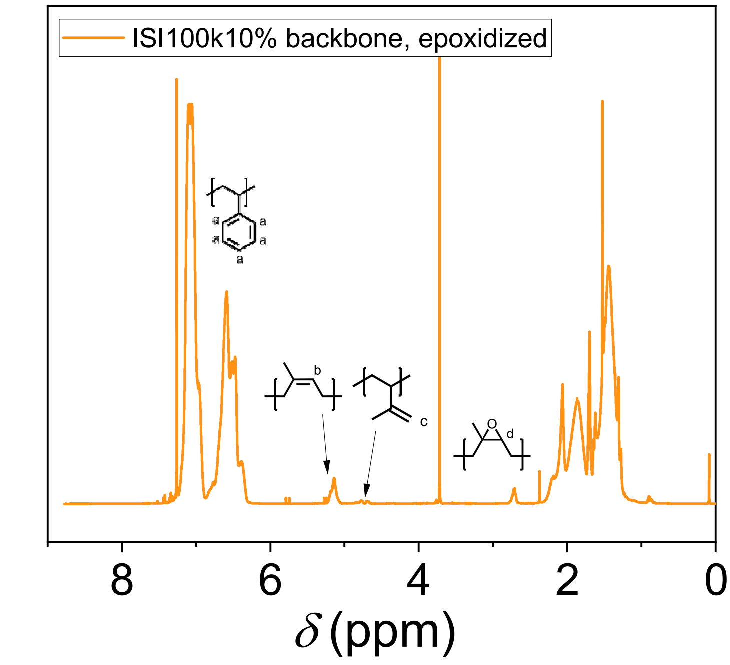

Supplementary Figure 19: ^{1}H NMR spectrum of an epoxidized ISI triblock of the exemplary synthesis above.

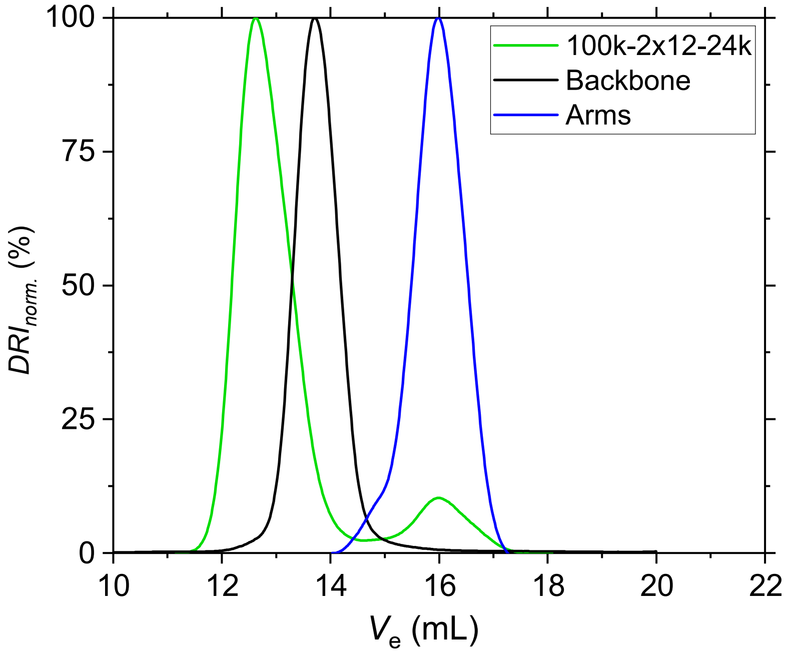

Supplementary Figure 20: Size exclusion chromatograms of the exemplary synthesis above.

MODELLING

The prediction of the elongational behavior of the pom-pom and the Doi-Edwards model was calculated using the pom-pom and the Doi-Edwards model in the IRIS Rheo-Hub software of Winter and Mours.[58,59] The molecular parameters of all samples were used as determined from

SEC and can be found in Supplementary Table 1. A plateau modulus of $G_0 = 2 \times 10^5$ Pa and a Rouse time of the entangled segments of $\tau_e = 0.036$ s ($T_{ref} = 140$ °C) was used for the simulation for all samples.[60]
